# *catena*-Poly[[bis­(2-chloro­pyrazine-κ*N* ^4^)cadmium]-di-μ-thio­cyanato-κ^2^ *N*:*S*;κ^2^ *S*:*N*]

**DOI:** 10.1107/S1600536813006338

**Published:** 2013-03-09

**Authors:** Susanne Wöhlert, Inke Jess, Christian Näther

**Affiliations:** a Institut für Anorganische Chemie, Christian-Albrechts-Universität Kiel, Max-Eyth-Strasse 2, 24118 Kiel, Germany

## Abstract

Reaction of cadmium thio­cyanate with 2-chloro­pyrazine leads to the polymeric title compound, [Cd(NCS)_2_(C_4_H_3_ClN_2_)_2_]_*n*_. The Cd^II^ cation, which is located on a center of inversion, is coordinated by two *N*-bonded and two *S*-bonded thio­cyanate anions and by two *N*-bonded 2-chloro­pyrazine ligands within a slightly distorted octa­hedron. The Cd^II^ cations are linked into chains along the *a* axis by bridging thio­cyanate anions.

## Related literature

For the background to this work and the synthesis of bridging thio­cyanato coordination polymers, see: Wöhlert *et al.* (2012 ▶, 2013 ▶).
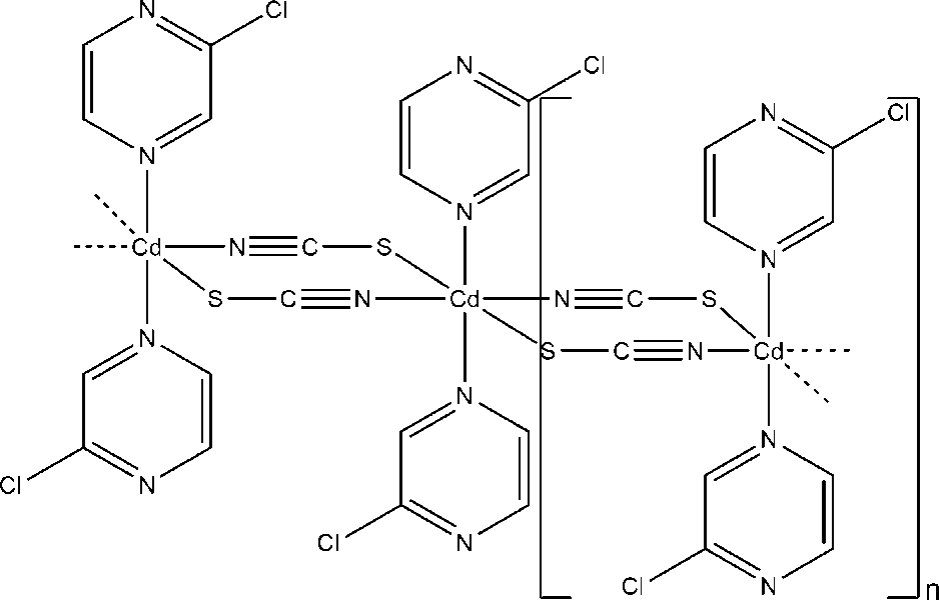


## Experimental

### 

#### Crystal data

[Cd(NCS)_2_(C_4_H_3_ClN_2_)_2_]
*M* *_r_* = 457.63
Triclinic, 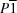

*a* = 5.7151 (6) Å
*b* = 6.7625 (8) Å
*c* = 11.2915 (13) Å
α = 76.895 (9)°
β = 82.639 (9)°
γ = 73.068 (8)°
*V* = 405.70 (8) Å^3^
*Z* = 1
Mo *K*α radiation
μ = 1.93 mm^−1^
*T* = 293 K
0.17 × 0.15 × 0.10 mm

#### Data collection

Stoe IPDS-2 diffractometer
Absorption correction: numerical (*X-SHAPE* and *X-RED32*; Stoe & Cie, 2008 ▶) *T*_min_ = 0.551, *T*_max_ = 0.719
5777 measured reflections
1592 independent reflections
1492 reflections with *I* > 2σ(*I*)
*R*_int_ = 0.068

#### Refinement

*R*[*F* ^2^ > 2σ(*F* ^2^)] = 0.029
*wR*(*F* ^2^) = 0.072
*S* = 1.02
1592 reflections
97 parameters
H-atom parameters constrained
Δρ_max_ = 0.56 e Å^−3^
Δρ_min_ = −0.49 e Å^−3^

### 

Data collection: *X-AREA* (Stoe & Cie, 2008 ▶); cell refinement: *X-AREA*; data reduction: *X-AREA*; program(s) used to solve structure: *SHELXS97* (Sheldrick, 2008 ▶); program(s) used to refine structure: *SHELXL97* (Sheldrick, 2008 ▶); molecular graphics: *XP* in *SHELXTL* (Sheldrick, 2008 ▶) and *DIAMOND* (Brandenburg, 2011 ▶); software used to prepare material for publication: *XCIF* in *SHELXTL* and *publCIF* (Westrip, 2010 ▶).

## Supplementary Material

Crystal structure: contains datablock(s) I, global. DOI: 10.1107/S1600536813006338/bt6896sup1.cif

Structure factors: contains datablock(s) I. DOI: 10.1107/S1600536813006338/bt6896Isup2.hkl

Additional supplementary materials:  crystallographic information; 3D view; checkCIF report

## Figures and Tables

**Table 1:** Selected bond lengths (Å)

Cd1—N1	2.287 (3)
Cd1—N12	2.417 (2)
Cd1—S1^i^	2.7071 (9)

Symmetry code: (i) 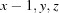.

**Fig. 1.**
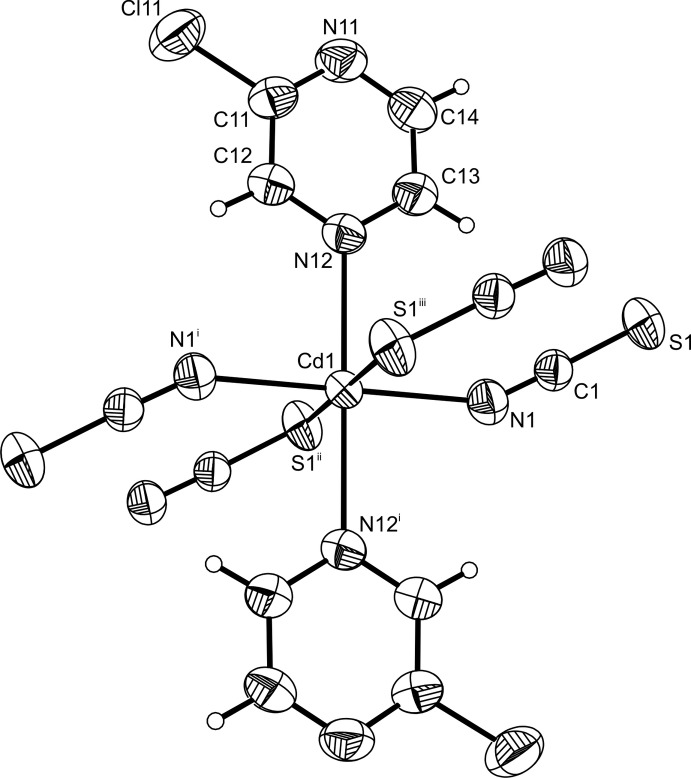
: Crystal structure of the title compound with labeling and displacement ellipsoids drawn at the 50% probability level. Symmetry code: i = -x + 1, -y + 1, -z; ii = x - 1, y, z; iii = -x + 2, -y + 1, -z.

**Fig. 2.**
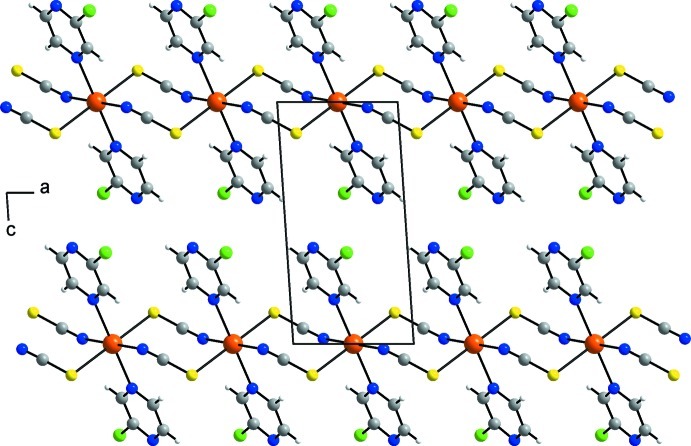
: Crystal structure of the title compound with view along the b-axis (orange = cadmium, blue = nitrogen, yellow = sulfur, green = chloro, grey = carbon, white = hydrogen).

## supplementary crystallographic information

### Comment

Recently we have reported on the synthesis and characterization of cadmium(II) thiocyanato coordination polymers with different neutral N-donor co-ligands. For [Cd(NCS)_2_(4-ethylpyridine)_2_]*_n_* a one-dimensional structure was observed, in which the metal cations are octahedrally coordinated by two N and two S atoms of the thiocyanato anions and two N atoms of the co-ligands all of them in an all-*trans* coordination (Wöhlert *et al.*, 2013). In contrast, for [Cd(NCS)_2_(pyridine)_2_]*_n_*, three different modifications were obtained in which the anionic ligands are all-*trans*, all-*cis* or *cis*-*cis*-*trans* coordinated (Wöhlert *et al.*, 2012). To investigate the influence of the neutral co-ligand in more detail, 2-chloropyrazine was selected, which can act as a bidentate as well as a monodentate ligand. Therefore, cadmium(II) thiocyanate was reacted with 2-chloropyrazine in water which results in the formation of single crystals suitable for structure determination.

The crystal structure of [Cd(NCS)_2_(2-chloropyrazine)_2_]*_n_* consists of cadmium(II) cations that are located on centers of inversion as well as of thiocyanato anions and 2-chloropyrazine ligand in general position. Each cadmium(II) cation is coordinated by two *N*-bonded and two *S*-bonded thiocyanato anions as well as two 2-chloropyrazine ligands within a slightly distorted octahedral geometry (Fig. 1). The CdN_4_S_2_ distances ranges from 2.287 (3) Å to 2.7071 (9) Å with angles arround the cadmium(II) cation between 87.75 (8) ° to 92.25 (8) ° and of 180 ° (Tab. 1). In the crystal structure the cadmium(II) cations are connected through *µ*-1,3 bridging thiocyanato anions into one-dimensional polymeric chains, in which all thiocyanato anions and the 2-chloropyrazine ligand are *trans*-coordinated (Fig. 2). The intrachain cadmium-cadmium separation amounts to 5.7151 (6) Å, whereas the shortest interchain Cd—Cd distance is 11.2915 (13) Å. This structural motiv is frequently found in cadmium thiocyanato coordination compounds and might represent the most stable coordination. However, in this context it is noted that for [Cd(NCS)_2_(pyridine)_2_]*_n_*, the form with a *cis*-*cis*-*trans* coordination represents the thermodynamic stable form at room-temperature and this might be an exception (Wöhlert *et al.*, 2012).

### Experimental

CdSO_4_x8/3H_2_O, Ba(NCS)_2_x3H_2_O and 2-chloropyrazine were obtained from Alfa Aesar. All chemicals were used without further purification. Cd(NCS)_2_ was prepared by the reaction of equimolar amounts of CdSO_4_x8/3H_2_O with Ba(NCS)_2_x3H_2_O in water. The resulting precipitate of BaSO_4_ were filtered off and the filtrate were concentrated to complete dryness resulting in white residues of Cd(NCS)_2_. The purity was checked by XRPD and CHNS analysis. 0.1 mmol (22.0 mg) Cd(NCS)_2_ and 0.4 mmol (32.0 µ*L*) 2-chloropyrazine were reacted in 1 ml water. Colorless single crystals of the title compound were obtained after three days.

### Refinement

All H atoms were located in difference map but were positioned with idealized geometry and were refined isotropic with *U*_iso_(H) = 1.2 *U*_eq_(C) using a riding model with C—H = 0.93 Å.

### Crystal data

**Table:**

[Cd(NCS)_2_(C_4_H_3_ClN_2_)_2_]	*Z* = 1
*M**_r_* = 457.63	*F*(000) = 222
Triclinic, *P*1	*D*_x_ = 1.873 Mg m^−^^3^
Hall symbol: -P 1	Mo *K*α radiation, λ = 0.71073 Å
*a* = 5.7151 (6) Å	Cell parameters from 5777 reflections
*b* = 6.7625 (8) Å	θ = 1.9–26.0°
*c* = 11.2915 (13) Å	µ = 1.93 mm^−^^1^
α = 76.895 (9)°	*T* = 293 K
β = 82.639 (9)°	Block, colourless
γ = 73.068 (8)°	0.17 × 0.15 × 0.10 mm
*V* = 405.70 (8) Å^3^	

### Data collection

**Table:**

Stoe IPDS-2 diffractometer	1592 independent reflections
Radiation source: fine-focus sealed tube	1492 reflections with *I* > 2σ(*I*)
Graphite monochromator	*R*_int_ = 0.068
ω scan	θ_max_ = 26.0°, θ_min_ = 1.9°
Absorption correction: numerical (*X-SHAPE* and *X-RED32*; Stoe & Cie, 2008)	*h* = −7→7
*T*_min_ = 0.551, *T*_max_ = 0.719	*k* = −8→8
5777 measured reflections	*l* = −13→13

### Refinement

**Table:**

Refinement on *F*^2^	Primary atom site location: structure-invariant direct methods
Least-squares matrix: full	Secondary atom site location: difference Fourier map
*R*[*F*^2^ > 2σ(*F*^2^)] = 0.029	Hydrogen site location: inferred from neighbouring sites
*wR*(*F*^2^) = 0.072	H-atom parameters constrained
*S* = 1.02	*w* = 1/[σ^2^(*F*_o_^2^) + (0.0411*P*)^2^] where *P* = (*F*_o_^2^ + 2*F*_c_^2^)/3
1592 reflections	(Δ/σ)_max_ < 0.001
97 parameters	Δρ_max_ = 0.56 e Å^−^^3^
0 restraints	Δρ_min_ = −0.49 e Å^−^^3^

### Special details

**Table:**

Geometry. All e.s.d.'s (except the e.s.d. in the dihedral angle between two l.s. planes) are estimated using the full covariance matrix. The cell e.s.d.'s are taken into account individually in the estimation of e.s.d.'s in distances, angles and torsion angles; correlations between e.s.d.'s in cell parameters are only used when they are defined by crystal symmetry. An approximate (isotropic) treatment of cell e.s.d.'s is used for estimating e.s.d.'s involving l.s. planes.
Refinement. Refinement of *F*^2^ against ALL reflections. The weighted *R*-factor *wR* and goodness of fit *S* are based on *F*^2^, conventional *R*-factors *R* are based on *F*, with *F* set to zero for negative *F*^2^. The threshold expression of *F*^2^ > σ(*F*^2^) is used only for calculating *R*-factors(gt) *etc*. and is not relevant to the choice of reflections for refinement. *R*-factors based on *F*^2^ are statistically about twice as large as those based on *F*, and *R*- factors based on ALL data will be even larger.

### Fractional atomic coordinates and isotropic or equivalent isotropic displacement parameters (Å^2^)

**Table:**

	*x*	*y*	*z*	*U*_iso_*/*U*_eq_	
Cd1	0.5000	0.5000	0.0000	0.04055 (12)	
N1	0.7466 (5)	0.7003 (4)	0.0217 (3)	0.0512 (6)	
N12	0.6360 (5)	0.2497 (4)	0.1840 (2)	0.0457 (6)	
C12	0.5473 (6)	0.0818 (5)	0.2234 (3)	0.0485 (7)	
H12	0.4340	0.0591	0.1794	0.058*	
C11	0.6252 (7)	−0.0574 (5)	0.3298 (3)	0.0502 (8)	
C14	0.8718 (8)	0.1294 (6)	0.3560 (3)	0.0623 (9)	
H14	0.9856	0.1502	0.4005	0.075*	
C13	0.8027 (7)	0.2699 (6)	0.2508 (3)	0.0545 (8)	
H13	0.8726	0.3819	0.2247	0.065*	
N11	0.7817 (7)	−0.0377 (5)	0.3977 (3)	0.0590 (7)	
Cl11	0.5080 (2)	−0.27337 (15)	0.37726 (10)	0.0755 (3)	
S1	1.14072 (16)	0.73906 (13)	0.12925 (8)	0.0535 (2)	
C1	0.9108 (6)	0.7182 (4)	0.0647 (3)	0.0404 (6)	

### Atomic displacement parameters (Å^2^)

**Table:**

	*U*^11^	*U*^22^	*U*^33^	*U*^12^	*U*^13^	*U*^23^
Cd1	0.03692 (19)	0.04198 (17)	0.04287 (19)	−0.01361 (12)	−0.01125 (13)	−0.00014 (12)
N1	0.0460 (16)	0.0474 (13)	0.0632 (17)	−0.0154 (12)	−0.0118 (13)	−0.0089 (12)
N12	0.0451 (15)	0.0484 (13)	0.0416 (14)	−0.0117 (11)	−0.0087 (11)	−0.0031 (11)
C12	0.0487 (18)	0.0482 (15)	0.0459 (16)	−0.0095 (13)	−0.0086 (14)	−0.0057 (13)
C11	0.060 (2)	0.0455 (15)	0.0418 (16)	−0.0124 (14)	−0.0037 (15)	−0.0052 (12)
C14	0.071 (3)	0.071 (2)	0.0480 (18)	−0.0249 (19)	−0.0237 (18)	−0.0015 (16)
C13	0.059 (2)	0.0546 (17)	0.0511 (19)	−0.0204 (15)	−0.0145 (16)	−0.0011 (14)
N11	0.072 (2)	0.0601 (16)	0.0431 (14)	−0.0183 (15)	−0.0143 (14)	0.0004 (12)
Cl11	0.1033 (9)	0.0591 (5)	0.0665 (6)	−0.0365 (5)	−0.0086 (6)	0.0031 (4)
S1	0.0426 (5)	0.0638 (5)	0.0620 (5)	−0.0142 (4)	−0.0100 (4)	−0.0250 (4)
C1	0.0381 (16)	0.0394 (13)	0.0452 (16)	−0.0123 (11)	−0.0033 (13)	−0.0088 (12)

### Geometric parameters (Å, º)

**Table:**

Cd1—N1	2.287 (3)	C12—H12	0.9300
Cd1—N1^i^	2.287 (3)	C11—N11	1.303 (5)
Cd1—N12^i^	2.417 (2)	C11—Cl11	1.730 (3)
Cd1—N12	2.417 (2)	C14—N11	1.341 (5)
Cd1—S1^ii^	2.7071 (9)	C14—C13	1.364 (5)
Cd1—S1^iii^	2.7071 (9)	C14—H14	0.9300
N1—C1	1.158 (4)	C13—H13	0.9300
N12—C12	1.338 (4)	S1—C1	1.636 (3)
N12—C13	1.342 (4)	S1—Cd1^iv^	2.7071 (9)
C12—C11	1.381 (4)		
			
N1—Cd1—N1^i^	180.0	C12—N12—Cd1	120.7 (2)
N1—Cd1—N12^i^	88.62 (10)	C13—N12—Cd1	122.4 (2)
N1^i^—Cd1—N12^i^	91.38 (10)	N12—C12—C11	119.6 (3)
N1—Cd1—N12	91.38 (10)	N12—C12—H12	120.2
N1^i^—Cd1—N12	88.62 (10)	C11—C12—H12	120.2
N12^i^—Cd1—N12	180.00 (15)	N11—C11—C12	124.5 (3)
N1—Cd1—S1^ii^	92.25 (8)	N11—C11—Cl11	117.2 (2)
N1^i^—Cd1—S1^ii^	87.75 (8)	C12—C11—Cl11	118.2 (3)
N12^i^—Cd1—S1^ii^	91.06 (7)	N11—C14—C13	122.5 (4)
N12—Cd1—S1^ii^	88.94 (7)	N11—C14—H14	118.7
N1—Cd1—S1^iii^	87.75 (8)	C13—C14—H14	118.7
N1^i^—Cd1—S1^iii^	92.25 (8)	N12—C13—C14	121.3 (3)
N12^i^—Cd1—S1^iii^	88.94 (7)	N12—C13—H13	119.4
N12—Cd1—S1^iii^	91.06 (7)	C14—C13—H13	119.4
S1^ii^—Cd1—S1^iii^	180.00 (4)	C11—N11—C14	115.1 (3)
C1—N1—Cd1	150.2 (2)	C1—S1—Cd1^iv^	96.53 (11)
C12—N12—C13	116.9 (3)	N1—C1—S1	178.3 (3)

Symmetry codes: (i) −*x*+1, −*y*+1, −*z*; (ii) −*x*+2, −*y*+1, −*z*; (iii) *x*−1, *y*, *z*; (iv) *x*+1, *y*, *z*.
